# Effect of collagen cross-linkers on dentin bond strength: A systematic review and network meta-analysis

**DOI:** 10.3389/fbioe.2022.1100894

**Published:** 2023-01-24

**Authors:** Huan Chen, Guangdi Sun, Huimin Wang, Shiyang Yu, Zilu Tian, Song Zhu

**Affiliations:** Department of Prosthodontics, School and Hospital of Stomatology, Jilin University, Changchun, China

**Keywords:** cross-linkers, dentin adhesives, bonding performance, network meta-analysis, systematic review

## Abstract

**Objective:** This study aimed to evaluate the role of collagen cross-linkers in the bonding performance of the resin-dentin interface through a systematic review and a network meta-analysis.

**Sources:** The literature search was conducted in several databases like PubMed, EMBASE, Cochrane, Scopus and Web of Science from their inception till 30 April 2022.

**Study selection:** The inclusion criteria consisted of *in vitro* studies evaluating the micro-tensile and micro-shear bond strengths of different cross-linkers acting on dentin. Bayesian network meta-analysis was conducted using RStudio.

**Data:** Out of the 294 studies evaluated in the full-text analysis, 40 were included in the systematic review and meta-analysis. Most studies have used cross-linkers as primer (65.1%), followed by incorporating them into in adhesives and acid etching agents. The application methods of the adhesive system were classified as “etch-and-rinse (ER) adhesives” (77%) and “self-etching (SE) adhesives”. Moreover, there were six types of cross-linkers in this presented review, of which the most numerous were polyphenols.

**Conclusion:** Different application methods of cross-linkers, the long-term results showed that were only effective when used for longer durations, the immediate results were not statistically different. According to immediate and long-term results, etch-and-rinse (ER) adhesives showed a greater bonding performance than the control groups (*p* ≤ 0.05), whereas self-etching (SE) adhesives showed similar bond strength values (*p* ≥ 0.05). The result of network meta-analysis (NMA) showed that Dope like compound showed higher long-term bonding performance than other cross-linkers.

**Clinical significance**: Long-term clinical studies may be needed to determine the effect of the cross-linkers on the bonding properties.

## Introduction

Precise adhesion between resin composites and dentin substrate requires the infiltration of resin monomers into the demineralized dentin matrix after the partial dissolution of the mineralized layer (Matuda et al., 2016). Recent advancements in adhesive bonding techniques have resulted in the use of resin composite materials for dentin substrates, which includes collagen fibers, non-collagenous proteins, and carbonate apatite. The resin composite materials used in restorative procedures are made up of collagen, non-collagen and carbonate apatite. To support the tissues, the C-terminal and globular N-terminal propeptidesand non-separable terminal peptides of collagen accommodate hydroxyapatite crystals (Tjäderhane et al., 2013). Moreover, the infiltration of adhesive monomers to demineralized dentin creates a hybrid layer (HL), which remains the weakest region of adhesively-based restorations (Comba et al., 2020).

The goal of excellent adhesion is to achieve an effective resin-dentin interface, that is, stable, and provides good retention, marginal integrity, and clinical durability (Tjäderhane, 2015). Despite these advances, HL created on the variable organic dentin phase is imperfect and may degrade over time, leading to marginal discoloration, nanoleakage, and decreased composite retention (Mjör et al., 2002; Maravic et al., 2017). Several *in vitro* and *in vivo* studies have tried to elucidate the potential causes resin-dentin adhesion progression. Therefore, the most likely contributors to interfacial degradation are a hydrolytic breakdown of the polymerized resin compounds and endogenous protease-initiated degradation of the demineralized dentin collagen matrix (Hashimoto et al., 2000; Armstrong et al., 2004; Mazzoni et al., 2018; Comba et al., 2019). However, 1 year of water storage resulted in a significant fall in bond strength of 31%–70% (Hashimoto et al., 2000; Mazzoni et al., 2013a). Therefore, different strategies have been proposed to minimize the degradation of HL over time, such as enhancing collagen fibrils within HL, inactivating endogenous enzymes, or combining the two strategies (Breschi et al., 2008; Mazzoni et al., 2013b; Bedran-Russo et al., 2014).

Cross-linking of dentin matrix collagen is a naturally occurring mechanism in dentin that provides tensile strength (Bedran-Russo et al., 2008; Bedran-Russo et al., 2014). Hence, collagen cross-linkers were introduced as an alternative dentin pre-treatment to improve the durability of dentin-resin bonds (Fawzy et al., 2012; Fawzy et al., 2013). Furthermore, they enhance the collagen fibrils network by inducing intra and intermolecular cross-linking by several mechanisms. Firstly, cross-linking agents increase collagen structural architecture and inactivate the catalytic site of these enzymes, thus, disabling the access and posterior hydrolysis of collagenases (Hass et al., 2016a). Secondly, the use of competitive (or non-competitive) enzyme inhibitors creates an enzyme-substrate complex that prevents the hydrolysis of the collagen substrate (Baena et al., 2020; Chen et al., 2021).

Cross-linking agents, like adhesives, increase the hardness of the HL collagen matrix as improved biomechanical properties of the dentin matrix increase resin-dentin bond durability (Cai et al., 2018). A previous meta-analysis evaluating the effects of plant extracts as a primer on dentin bonding strength demonstrated an improvement in the immediate bond strength of adhesive (Zhao et al., 2022). Nevertheless, whether collagen cross-linkers can provide stable and long-lasting bonding strength to the adhesive interface is still debatable.

Therefore, this study aimed to systematically review all *in vitro* studies that assessed the role of collagen cross-linkers in the bonding performance of the resin-dentin interface immediately and for longer durations. The null hypothesis was that there would be no difference in bond strength values when the collagen cross-linkers were used in the bonding procedures.

## Materials and methods

Registered with PROSPERO (CRD42022365877), this study was conducted following updated guidelines for the PRISMA 2020 Statement: Systematic Review Reports (Page et al., 2021); the research question was “Can collagen cross-linkers improve the bonding performance of resin-dentin interface?”

### Literature search and information sources

The literature search strategy consisted of the following terms: cross-linkers, adhesives, and bond strength, as detailed in Supplementary Material S1. Two independent reviewers performed the literature search and screened five electronic databases (PubMed, EMBASE, Cochrane library, Scopus, and Web of Science) to identify relevant manuscripts that could be included. The database search was extended until 30 April 3022, because no publication year or language restrictions were used. Additionally, the reviewers also manually searched the reference lists of the collected manuscripts for additional relevant studies that met the inclusion criteria.

### Inclusion criteria

Inclusion criteria were *in vitro* or *ex vivo* studies evaluating the effects of cross-linkers on dentin and *in vitro* studies assessing immediate and long-term bond strengths, in the experimental group and control groups treated with cross-linkers and without them, respectively. Micro-tensile and micro-shear bond strengths (MTBS and MSBS) of adhesives (unit: MPa) studies, as well as using caries-affected or sound teeth. Studies focusing on deciduous and material-based substrates (e.g., resin composites, ceramics, metals), were excluded; studies lacking substrate data were not available after at least two email requests to the authors. The summary study design (PICOS) was as follows: P, dental adhesive; I, cross-linkers; C, without cross-linkers; O, bond strength.

### Study selection and data extraction

Duplicate records were removed after importing the articles into EndNoteX9 (Thomson Reuters); in case of any disagreement, a third review (SZ) was recruited to reach a consensus. Two researchers (HC and DS) extracted data independently using Microsoft Office Excel 2013 spreadsheets (Microsoft Corporation, Redmond, WA, United States) and tabulated relevant data. The following datas were extracted: study (year of publication), type of aging, dental adhesives used, cross-linkers used, and additional tests performed, such as scanning electron microscopy (SEM) evaluation. The corresponding authors were contacted *via* email to retrieve missing data of specific bond strength values and display results graphically or numerically or any other information.

### Quality assessment

Adapted from a previous study (Montagner et al., 2014), the quality assessment was done by two investigators based on the following parameters: teeth randomization, teeth free of caries/restoration, materials used according to manufacturers’ instructions, adhesive procedures performed by a single operator, sample size calculation, and operation blinding. The study was given a “Y” if the parameter was included and performed appropriately and an “N” if the parameter was missing or inadequately performed. The number of parameters that scored “Y”, 1 or 2 indicated a high risk, 3 to 3 medium, and 5 to 6 indicated a low risk of bias. Any differences between the two investigators were resolved by interviewing the third investigator.

### Statistical analysis

Meta-analyses were performed using Review Manager software version 5.3.5 (Nordic Cochrane Center, Cochrane Collaboration, Copenhagen, Denmark). The pooled effect estimates were derived using a random-effects model that compared the mean difference between bond strength values. The studies were divided into three categories based on how cross-linkers were used: (Matuda et al., 2016): applied to dentin as a pre-treatment solution that remains in contact with the surface (commonly used), (Tjäderhane et al., 2013), incorporated within the adhesive system, or (Comba et al., 2020) incorporated into the acid etching agent that is rinsed away from the surface. The adhesive system’s application methods were classified as “etch-and-rinse (ER) adhesives” and “self-etching (SE) adhesives”. It is worth noting that ER mode of universal adhesive is assigned to ER adhesives and the SE mode is assigned to SE adhesives. A comprehensive effect estimate was obtained by comparing standardized average differences between bond strength values of the experiment and the control groups. Furthermore, studies evaluating samples before and after the long-term process were analyzed separately. All *p*-values < 0.05 were considered statistically significant. Statistical heterogeneity of treatment effect among studies was assessed using the Cochran Q test and the inconsistent I^2^ test.

Bayesian network meta-analysis (NMA) was performed on bond strength data of various cross-linkers, and it was classified as 1) control; 2) aldehydes, including glutaraldehyde (GA), acrylic primer, 4-formylphenyl acrylate (FA); 3) 1-Ethyl-3-(3-dimethylaminopropyl) carbodiimide (EDC); 4) polyphenols, including grape seed extracts (main component: proanthocyanidins (PA), epigallocatechin-3-gallate (EGCG), quercetin, etc; 5) chitosan; 6) riboflavin; 7) Dope like compound such as mussel adhesive proteins (MAP) and dopamine methacrylamide (DMA). Additionally, Bayesian random effects pairwise and NMA were performed to derive pairwise, indirect and network estimates, one for immediate and the other for long-term bond strength. Separated analyses were conducted for immediate and long-term results. The JAGS program implemented in the R package *gemtc* 0.8–2 (Valkenhoef et al., 2012) evaluated Network plots and league tables using Bayesian random effects modeling and Markov chain Monte Carlo simulations (van Ravenzwaaij et al., 2018), with 20,000 iterations for adaptation. Bayesian random-effects NMA estimated the effect as mean difference (MD) with 95% credible intervals (95% CrI). A *p*-value < 0.05 was considered statistically significant. Additionally, the average ranking and cumulative ranking curves (SUCRA) were used to rank groups (Salanti et al., 2011), which were shown graphically, and the pairs or values were generated from the NMA table (Rouse et al., 2017).

## Results

### Search strategy

**FIGURE 1:**
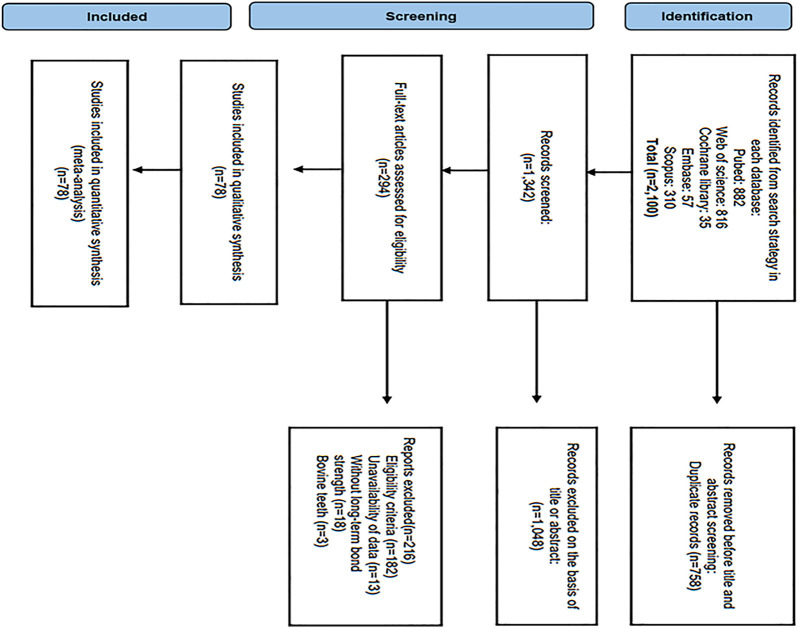
PRISMA flow chart of study selection.

From 2,100 potentially eligible studies, 294 and 78 were selected for full-text analysis, and the systematic review, respectively (Figure 1). A total of 216 studies were not included; many studies were excluded based on the eligibility criteria (182); data not available (Mazzoni et al., 2013b); without long-term bond strength (Hass et al., 2016a), and conducted on bovine teeth (Comba et al., 2020). Finally, seven-eight studies were included in the review (Cova et al., 2011; Castellan et al., 2013; Chiang et al., 2013; Andre et al., 2015; Carvalho et al., 2016; Abunawareg et al., 2017; Bacelar-Sa et al., 2017; Albuquerque et al., 2019; Costa et al., 2019; Czech et al., 2019; Baena et al., 2020; Beck and Ilie, 2020; Comba et al., 2020; Abdelshafi et al., 2021; Baldion et al., 2021; Chen et al., 2021; Dacoreggio et al., 2021; Abd El-Aal et al., 2022; Beck and Ilie, 2022) and meta-analysis for which the primary data (mean bond strength, standard deviation, and the number of test samples) could be retrieved.

### Descriptive analysis

All studies included in the review were published between 2011 and 2022. Cross-linkers were applied as a pre-treatment solution in the majority of studies (65.1%), followed by incorporation into adhesive systems (30.1%), and only four studies (4.8%) added them into the acid etching agent to evaluate bonding performance (Table 1). Concerning the resin composites used to prepare restorations, the most commonly used materials are purchased from the 3M ESPE industry (e.g., Filtek Z250, Filtek Z350, Filtek Supreme, and Filtek P60 (3M ESPE)), followed by Kulzer (Charisma), FGM (Opallis), VOCO (Grandio) and Kuraray (Clearfil A-PX), Ivoclar-Vivadent (Tetric Ceram) and Ivoclar-Vivadent (Bluephase). The majority of studies (76.9%) applied a #600-grit SiC abrasive paper at the dentin surface before applying adhesive In contrast, others used grit sizes ranging from #180-grit to #1200-grit SiC or a sequence of SiC at varying final grits.

**TABLE 1:** Main characteristics of the *in vitro* studies included in the review.

Study (year)	Type of aging	Dental adhesives used	Cross-linkers used	Additional tests performed	Bond strength test used
**1. Data from included studies that used cross-linkers as a pre-treatment solution**					
Abd El-Aal et al. (2022)	After storage in artificlial saliva for 24 h, 6 months	Single Bond Universal (3M ESPE)	10% Proanthocyanidin Grape Seed Extract (GSE) solution	Scanning electron microscopy (SEM)	ΜTBS
Abdelshafi et al. (2021)	Thermocycling for 10,000 cycles at 5°C and 55 °C	Single Bond Universal (3M ESPE)	6.5% GSE.	SEM.	ΜTBS
Abunawareg et al. (2017)	After storage in distilled water for 24 h, 6 months and 12 months	Adper Single Bond 2 (3M ESPE)	0.5 M EDC-HCl, 1 wt% riboflavin-5-monophosphate sodium salt hydrate	Nanoleakage evaluation	ΜTBS
Bacelar-Sa et al. (2017)	After storage in artificlial saliva for 24 h, 6 months	G-Aenial (GC Corp), All-Bond 3 (Bisco Inc.), Scotchbond Universal (3M ESPE), Prime & Bond Elect (Dentsply Caulk)	5% glutaraldehyde (GA),6.5% proanthocyanidin-rich grape seed extract	Dentin Sealing	ΜTBS
Baena et al. (2020)	Thermocycling for 10,000 cycles at 5°C and 55 °C	Optibond FL (Kerr), Scotchbond Universal (3M ESPE)	0.1% chitosan solution	Nanoleakage, zymography	ΜTBS
Baldion et al. (2021)	After storage in artificial saliva at 37 °C for 24 h, 18 months	Adper Single Bond 2 (3M ESPE)	GA, myricetin (MYR), proanthocyanidins (PA)	Failure mode	ΜTBS
Beck and Ilie, (2020)	After 1 week, 1 month, 3 months, 6 months, and 1 year of distilled water at 37°C	Clearfil SE Bond (Kuraray)	Epigallocatechin-3-gallate (EGCG)PA.	Failure mode	ΜΜSBS
Beck and Ilie, (2022)	After 1 week, 1 month, 3 months, 6 months, and 1 year of immersion duration (distilled water, 37°C)	Clearfil SE Bond (Kuraray)	Riboflavin (RF)	Failure mode	ΜΜSBS
Carvalho et al. (2016)	After storage in water for 24 h, 6 months	Adper Single Bond 2 (3M ESPE)	2% EGCG.	Failure mode	ΜTBS
Castellan et al. (2013)	After storage in artificial saliva at 37 °C for 24 h, 6,12 months	Adper Single Bond Plus (3M ESPE), One Step Plus (Bisco)	6.5% GSE.	Failure mode	ΜTBS
Chen et al. (2021)	Thermocycling for 10,000 cycles at 5°C and 55 °C	Adper Single Bond Plus (3M ESPE)	1-ethyl-3-(3-dimethylaminopropyl) carbodiimide (EDC)	*In situ* zymography, inhibition of collagenase activity, evaluation of the micropermeability and nanoleakage	ΜTBS
Chiang et al. (2013)	Thermocycling for 5,000 cycles at 5°C and 60 °C	Scotchbond Multi-purpose adhesive (3M ESPE)	Glutaraldehyde and different RF/UVA protocols	Gel electrophoresis analysis, nanoindentation test, evaluation of nanoleakage and morphology of hybrid layer	ΜTBS
Comba et al. (2020)	After 24 h, 1 year of aging in artificial saliva at 37°C	Scotchbond Universal (3M ESPE)	N,N′-dicyclohexylcarbodiimide (DCC)	Nanoleakage expression, *in situ* zymography	ΜTBS
Costa et al. (2019)	After storage in distilled water at 37°C for 24 h, 6 months	Clearfil SE Bond (Kuraray)	0.1% aqueous EGCG solution	Failure mode	ΜTBS
Cova et al. (2011)	After storage in distilled water at 37°C for 24 h, 6,12 months	XP Bond adhesive (Dentsply)	0.1% RF and exposed to UVA for 2 min	Nanoleakage expression, zymographic analysis	ΜTBS
de Paula (2022)	1000 cycles; 30 s in 5°C and 30 s in 55°C with 10 s interval	Optibond S (Kerr)	6.5% PA, 2% cardanol, lignin at 1, 2 or 4% concentrations	Failure pattern, nanoleakage, micropermeability assay, *in situ* degree of conversion, elastic modulus	ΜTBS
Dávila-Sánchez et al. (2020)	After thermo-cycling aging (25,000 cycles)	Scotchbond Universal (3M Oral Care)	quercetin (QUE), hesperidin (HPN), rutin (RUT), naringin (NAR), or PA.	Nanohardness within adhesive layer, hybrid layer and dentin, confocal ultramorphology evaluation	ΜTBS
de Siqueira (2020)	After 2 years of aging in distilled water at 37°C	Prime&Bond Elect (Dentsply Sirona), Scotchbond Universal (3M Oral Care), Tetric N-Bond Universal (Ivoclar Vivadent)	6.5 wt% PA or with light-cure 0.1 wt% RF.	Evaluation of silver nitrate deposition, conversion of degree (DC) within, hybrid layer nanohardness and Young’s modulus	ΜTBS
Fang et al. (2017)	After thermo-cycling aging (2,500 cycles)	Gluma Comfort Bond (Heraeus Kulzer)	Mussel adhesive protein (MAP)	Inhibition of collagenase activity, inhibiting the degradation of demineralized dentin matrix	ΜTBS
Fawzy et al. (2012)	After 24 h and 4 months of storage in distilled water	Adper Single bond 2 (3M ESPE)	Photo-activation of RF by ultraviolet (UVA)	Ultimate tensile strength (UTS) and hydroxyproline (HYP) release	ΜTBS
Fawzy et al. (2013)	After 24 h and 6 months of storage in distilled water	Adper Single bond 2 (3M ESPE)	Chitosan/RF-modified solution	SEM investigation, Nanoindentation testing, UTS, HYP.	ΜTBS
Fernandes (2021)	After 24 h and 12 months of storage in artificial saliva	Clearfil SE Bond (Kuraray Medical)	EGCG	Nanoleakage at the adhesive interface	
Fernandes (2022)	Specimen were evaluated at 24 h and after 10,000 thermocycles	Single Bond Universal (3M), Clearfil Universal (Kuraray Noritake Dental), Ambar Universal (FGM), Clearfil SE Bond (Kuraray Noritake Dental)	EDC and DCC.	Failure mode	ΜTBS
Fialho (2019)	After 24 h and 6 months of storage in distilled water	Adper Single Bond 2 (3M ESPE)	0.02%,0.2% EGCG and 0.5% EGCG.	Fracture pattern analysis, analysis of nanoleakage at the adhesive interface	ΜTBS
Gerhardt (2016)	After storage in water for 24 h and 6 months	Clearfil SE Bond (Kuraray Medical)	EGCG	Failure pattern	ΜTBS
Hass et al. (2016b)	24 h, 18 months of storage in distilled water at 37°C	Single Bond Plus (3M ESPE), Tetric N-Bond (Ivoclar Vivadent)	6.5 wt% PA, UVA-activated 0.1 wt% RF, 5 wt% GA.	Nanoleakage evaluation, DC, *in situ* zymography, cytotoxicity evaluation	ΜTBS
Jowkar (2020)	After 24 h and 6 months of storage in distilled water	Adper Single Bond (3 M ESPE)	PA.	Failure mode	ΜΜSBS
Li (2017)	After storage for 24 h, or 1-month collagenase aging in the collagenase-containing artificial saliva	Adper Single Bond 2 (3M ESPE)	0.1, 0.5, or 1.0 wt% quercetin/ethanol agents	Interfacial nanoleakage evaluation, surface contact angle test, *in situ* zymography, antibacterial evaluation, cytotoxicity evaluation	ΜTBS
Li (2018)	After 24 h and 6 months of storage in artificial saliva at 37°C	Adper Single Bond 2 (3M ESPE)	GA, Baicalein	Failure mode, interfacial Nanoleakage Testing	ΜTBS
Li et al. (2021a)	After 10,000 thermocycles	Adper Single Bond 2 (3M ESPE)	N-(3,4-dihydroxyphenethyl) methacrylamide (DMA)	Failure mode, nanoleakage evaluation, *in situ* zymography, cytotoxicity test, DC, Derjaguin-Müller-Toporov (DMT) modulus	ΜTBS
Li et al. (2021b)	Thermocycling, 5°C–55°C, 10,000 times	Adper Single Bond 2 (3M ESPE)	Dopamine methacrylamide (DMA)	Fracture pattern, Interfacial nanoleakage evaluationDC.	ΜTBS
Li (2022)	After 24 h, 3, 6, and 12 months of storage	Adper Single Bond Plus (3M ESPE)	Glycol chitosan-EDTA.	Transmission electron microscopy (TEM)	ΜTBS
Maravic et al. (2018)	After 24 h and 1 year of storage in artificial saliva at 37°C	Adper Scotchbond 1XT (3M ESPE)	0.01% acrolein (ACR) aqueous solution	Zymography of dentine extracts, *in situ* zymography of resin-dentine interfaces	ΜTBS
Maravic et al. (2021)	After 24 h and 5 years of storage in artificial saliva at 37°C	XP Bond (Dentsply Sirona), Clearfil SE Bond (Kuraray-Noritake)	EDC.	*In Situ* Zymography, micro–Raman Spectroscopy	ΜTBS
Mazzoni (2013a)	After 24 h and 1 year of storage in artificial buffer at 37°C	Optibond FL (Kerr), Scotchbond 1XT (3 M ESPE)	EDC.	Interfacial nanoleakage evaluation, zymographic analysis	ΜTBS
Mazzoni et al. (2018)	After 24 h and 1 year of storage in artificial saliva at 37°C	Clearfil SE Bond (Kuraray Dental), XP Bond (Dentsply DeTrey GmbH)	EDC.	Zymographic analysis	ΜTBS
Neri (2016)	Distilled water at 37°C for 24 h, 6 months, and 12 months	Adper Easy One (3M ESPE)	EGCG	Failure modes	ΜTBS
Paik (2022)	Specimen were evaluated at 24 h and after 10,000 thermocycles	All-Bond Universal (BISCO Inc.)	Three flavonoids: icaritin (ICT), fisetin (FIS), silibinin (SIB)	Nanoleakage assessment, failure mode	ΜTBS
Paludo (2019)	The specimens for the 12-month group were stored in distilled water at 37 °C	Adper Single Bond 2 (3M ESPE)	GSE.	SEM, atomic force microscopy (AFM)	ΜTBS
Paschoini (2021)	24 h and 6 months of water storage	Adper Single Bond 2 (3M ESPE), Clearfil SE Bond (Kuraray)	Chitosan	SEM analyses	ΜTBS
Paulose (2017)	24 h or 1 year in distilled water at 37°C	Adper Single Bond 2 (3M ESPE), Single Bond Universal (3M ESPE)	GSE.	SEM evaluation, interfacial nanoleakage expression	ΜTBS
Paulose (2018)	24 h or 1 year in distilled water at 37°C	Adper scotchbond Multi-Purpose (3M ESPE), Single Bond Universal (3M ESPE)	EDC.	SEM evaluation, interfacial nanoleakage expression	ΜTBS
Porto (2018)	24 h or 120 days in distilled water at 37°C	Single Bond Universal (3M ESPE)	QUE, resveratrol	Fourier transform infrared analysis, collagenase treatment, fracture pattern evaluation	ΜTBS
Santiago (2013)	The remaining sticks were stored in 0.3 mMol/l sodium azide (pH 7.31) at 37 °C for 6 months	Adper Single Bond 2 (3M ESPE)	EGCG	Fracture modes	ΜTBS
Scheffel (2015)	Speciman were stored in artificial saliva at 37 °C for 24 h, 6 or 12 months	Adper Single Bond 2 (3M ESPE)	0.5 mol/L 1-ethyl-3-(3-dimethylaminopropyl) carbodiimide (EDC)	Nanoleakage analysis	ΜTBS
Singh et al. (2015)	Speciman were stored in artificial saliva at 37 °C for 24 h, 6 months	G-Bond (GC Corp.) and OptiBond-All-In-One (Kerr)	EDC.	Fracture Modes	ΜΜSBS
Sun (2018)	5,000 cycles between 5°C and 55 °C, with a dwell time of 20 s and a transfer time of 10s	Adper Single Bond 2 (3M ESPE)	EGCG	Confocal laser scanning microscopy (CLSM), failure mode	ΜTBS
Venigalla (2016)	After 24h, 6 months storage in artificial saliva	Adper Single Bond (3M ESPE)	UVA-activated 0.1% RF, 1 M EDC, and 6.5 wt% PA.	Failure mode	ΜTBS
Yang (2016)	After 24 h water storage or 10,000 runs of thermocycling	Adper Single Bond 2 (3M ESPE)	EGCG	Fracture mode, nanoleakage evaluation	ΜTBS
Yu et al. (2022)	Specimen were evaluated at 24 h and after 10,000 thermocycles	Single Bond Universal (3M ESPE) and All Bond Universal (Bisco)	4-formylphenyl acrylate (FA)	DC, contact angle, evaluation of the bonded interface and CLSM.	ΜTBS
Zhang (2014)	After storage in artificial saliva for 0, 3 and 6 months	Adper Single Bond 2 (3M ESPE)	EDC.	Failure modes, HYP.	ΜTBS
Zhang (2016)	0.9% NaCl solution at 37°C for 24 h and 90 days	Adper Single Bond 2 (3M ESPE)	EDC.	Fracture modes, SEM evaluation, resistance against enzymatic degradation test	ΜTBS
Zheng and Chen, (2017)	After 24h, 3 months	Adper Single Bond 2 (3M ESPE)	PA	Micro permeability and MMP substrate activity	ΜTBS
**2. Data from included studies that used cross-linkers incorporated into the adhesive system**					
Albuquerque et al. (2019)	After storage in distilled water at 37°C for 24 h, 6,12 months	Adper Single Bond 2 (3M ESPE)	EGCG	Failure mode	ΜTBS
Andre et al. (2015)	After storage in artificlial saliva for 1 week, 1 year	Gluma Comfort Bond (Heraeus Kulzer GmbH), Gluma 2 Bond (Heraeus Kulzer GmbH)	GA	Failure mode and facultative bacteria	ΜTBS
Beck and Ilie, 2020	After 1 week, 1 month, 3 months, 6 months, and 1 year of immersion duration (distilled water, 37°C)	Clearfil SE Bond 2	EGCG, PA, HPN.	Failure mode	ΜΜSBS
Beck and Ilie, (2022)	After 1 week, 1 month, 3 months, 6 months, and 1 year of immersion duration (distilled water, 37°C)	Clearfil SE Bond 2	Riboflavin	Failure mode	ΜΜSBS
Czech et al. (2019)	After storage in water for 24 h, 6 months and 12 months	Adper Single Bond 2 (3M ESPE)	EGCG solution (200 μg/ml)	Micromorphological evaluation, flexural strength	ΜTBS
Dacoreggio et al. (2021)	After 24 h and 6 months of storage in artificial saliva	Scotchbond Universal (3 M ESPE)	0.5% chitosan	Failure mode analysis, hybrid layer micromorphological analysis, *in situ* zymography	ΜTBS
Daood et al. (2018)	After 24 h and 36 months of storage in artificial saliva	The experimental two-step etch-and-rinse adhesives	Chitosan-Riboflavin modified adhesive	Failure mode, cell viability	ΜTBS
Daood et al. (2018)	After 24 h and 12 months in artificial saliva	The experimental two-step etch-and-rinse adhesives	Adhesives modified with (m/m, 0, 1%, 2% and 3% ribose)	MMP-2 and cathepsin K specimen preparation and activities, TEM investigation, micro-Raman spectroscopy, Cell viability	ΜTBS
Daood (2020a)	After 24 h and 12 months of storage in artificial saliva	The experimental universal adhesives	Riboflavin and D-Alpha 1000 Succinate polyethylene (VE-TPGS) incorporated in experimental adhesive systems	SEM resin-dentine interface, TEM of resin dentine adhesive, Cytotoxicity, MMP profilometry, Intermolecular measurement simulation, Molecular docking simulations	ΜTBS
Daood (2020b)	Specimen were evaluated at 24 h and after 10,000 thermocycles	The experimental universal adhesives	A novel dentin adhesive modified with both quaternary ammonium (QA) and riboflavin (RF) compounds (QARF)	SEM, nano-leakage expression, nano-Computerized Tomography (Nano CT), micro-raman depth analysis, confocal analysis: biofilm viability, MMPs and Cathepsin-K expressions	ΜTBS
de Macedo (2019)	After 24 h,6 months of storage in distilled water at 37°C	The experimental adhesives	EGCG	Degree of conversion, flexural strength and modulus	ΜTBS
Diolosà et al. (2014)	Thermocycling for 6,000 cycles at 5°C and 55 °C	The experimental three-step etch-and-rinse adhesives	Modification primer of chitosan with methacrylic acid (Chit-MA)	Interfacial Nanoleakage Analysis, Confocal Laser Scanning Microscopy (CLSM)	ΜTBS
dos Santos (2018)	24 h and 6 months stored in deionized water at 37°C	The experimental adhesives	chitosan-methacrylate (Chit-MA)	Failure mode, antimicrobial test	ΜTBS
Du (2012)	After 24h, 6 months of storage in distilled water at 37°C	Adper Single Bond 2 (3M ESPE)	EGCG was incorporated at a ratio of 100, 200, and 300 mg/ml into a dental adhesive	Antibacterial effect test, degree of conversion	ΜTBS
Epasinghe (2015)	After storage in artificial saliva for 24 h and 6 months	The experimental adhesives	PA	Fracture pattern analysis, nanolaekage evaluation	ΜTBS
Fernandes (2021)	After 24 h and 12 months of storage in artificial saliva	Clearfil SE Bond (Kuraray Medical)	EGCG	Nanoleakage at the adhesive interface	
Fonseca et al. (2019)	After 24 h and 6 months of storage in distilled water	The experimental adhesives	EGCG	Failure mode, SEM evaluation	ΜTBS
Fu et al. (2020)	After storage in artificial saliva for 1 week and 6 months	Scotch bond Universal (3M ESPE), UA Zipbond (SDI)	0.1% riboflavin-5-phosphate modified adhesive	Hydroxyproline release, evaluation of dentin apparent elastic modulus, determination of the crosslinking degree, SEM evaluation, circular dichroism, docking simulation	ΜTBS
Gotti (2015)	After storage in water for 24 h and 6 months	Adper Single Bond 2 (3M ESPE), Clearfil SE Bond (Kuraray Medical), Adper Easy Bond (3M ESPE)	vitamin C, vitamin E and quercetin	Failure pattern, interfacial nanoleakage	ΜTBS
Hechler (2012)	0, 1,4, 26, and 52- week in buffer or collagenase solution	The experimental adhesives	PA.	SEM.	ΜTBS
Lee (2017)	After 24 h and 6 months of storage in distilled water	Comfort Bond, Comfort Bond & Desensitizer	GA.	Collagen solubilization and mass loss, HYP.	ΜTBS
Magalhaes Rolim (2022)	After 24 h, 12 months of storage in distilled water at 37°C	Ambar Universal (FGM), Clearfil SE Bond (Kuraray Dental)	Incorporating the concentration of PA 1.0 wt% or EGCG 1.0 wt% into two adhesive systems with a self-etch approach	Nanoleakage evaluation, *in situ* zymography	ΜTBS
Yang (2017)	After storage in deionized water for 24 h, or 1-month collagenase aging in the prepared 0.1 mg/ml collagenase-containing artificial saliva in the dark at 37 °C	Adper Single Bond 2 (3M ESPE)	Incorporating quercetin into a commercial adhesive at three concentrations (100, 500 and 1000 μg/ml)	Interfacial nanoleakage evaluation, *in situ* zymography	ΜTBS
Yu et al. (2017)	After 24 h and 5000 thermocycles	Adper Single Bond 2 (3M ESPE)	EGCG	Antibacterial test, SEM, CLSM, and Micro-Raman analysis, inhibition of dentin-originated collagen proteases activities	ΜTBS
Zhao 2021	After 24 h, 6 months storage in artificial saliva	Adper Single Bond 2 (3M ESPE)	Urushiol derivative	Failure modes, nanoleakage	ΜTBS
**3. Data from included studies that used cross-linkers incorporated into acid etching agent**					
De-Paula (2020)	Thermocycling for 1,000 cycles at 5°C and 55 °C	Optibond S (Kerr)	: Lignin (LIG) from industrial paper production residue, Cardanol (CARD) from cashew-nut shell liquid, and PA.	FTIR spectroscopy, fracture mode, nanoleakage assessment	ΜTBS
Hass et al. (2016a)	After 24 h and 6 months of storage in distilled water	Adper Single Bond Plus (3M ESPE)	2% PA-containing 10% phosphoric acid	Nanoleakage evaluation, *in situ* zymography by CLSM, resin–enamel micro-shear bond strength	ΜTBS
Loguercio et al. (2017)	After 24h, 1 year of storage in distilled water at 37°C	Adper Single Bond 2 (3M ESPE)	PA.	Fracture pattern, nanoleakage evaluation	ΜTBS
Paludo (2019)	The specimens for the 12-month group were stored in distilled water at 37 °C	Adper Single Bond 2 (3M ESPE)	GSE	SEM, atomic force microscopy (AFM)	ΜTBS

**FIGURE 2:**
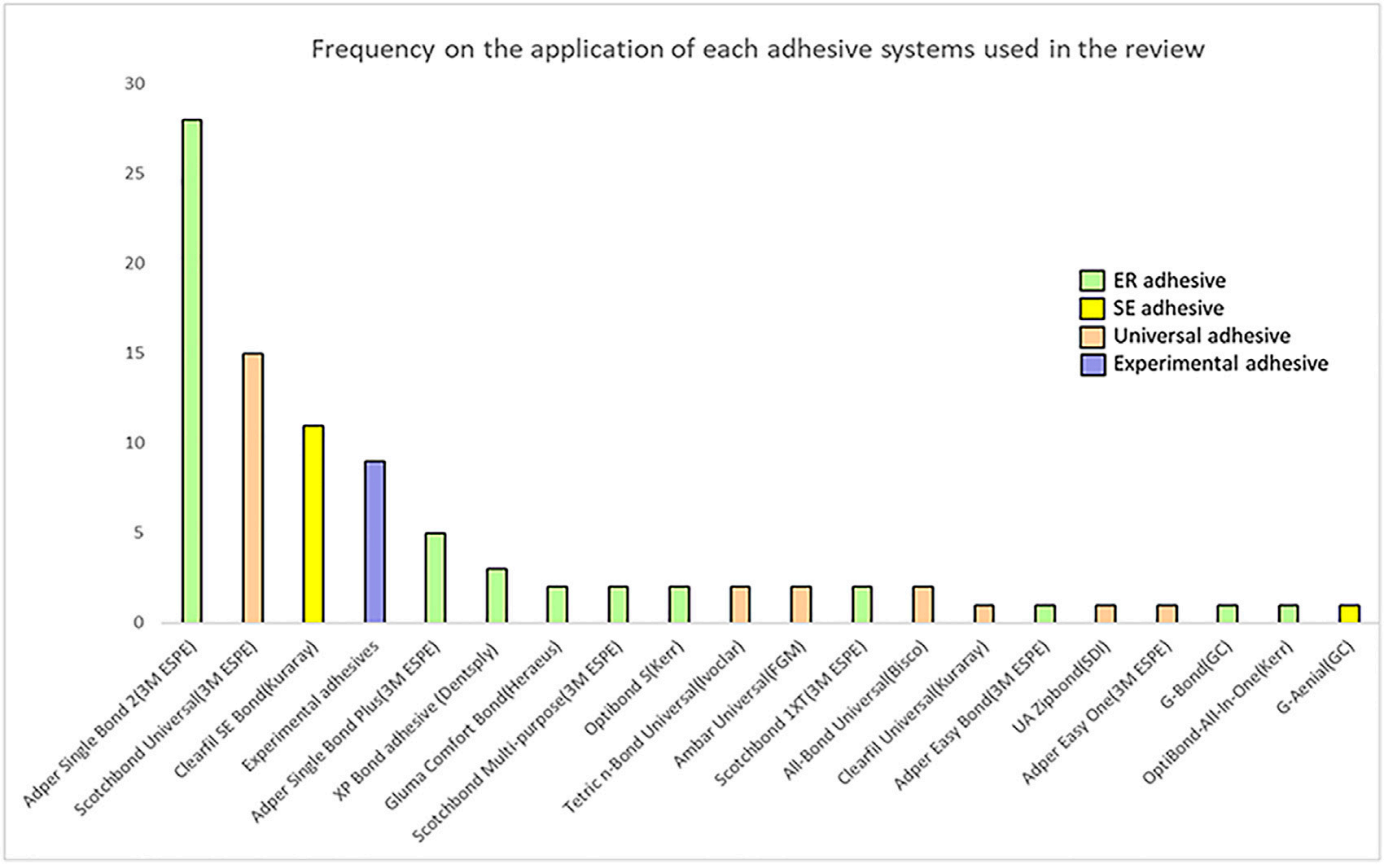
Frequency on the application of each adhesive systems used in the review.

Figure 2 depicts the adhesive systems used in this review, which were allocated according to their application strategies. Although Single Bond Universal (3M ESPE) and Adper Single Bond 2 (3M ESPE) were the most frequently used adhesives, eight kinds of universal adhesives were utilized in total: Single Bond Universal (3M ESPE), All Bond Universal (3M ESPE), Tetric N-Bond Universal (Ivoclar), Ambar Universal (FGM), Clearfil Universal (Kuraray), G-Bond (GC Corp), OptiBond-All-In-One (Kerr) and UA Zipbond (SDI), while ER adhesives included Adper Single Bond 2 (3M ESPE), Adper Single Bond Plus (3M ESPE), XP Bond Adhesive (Dentsply), Adper Scotchbond 1XT (3M ESPE), Prime and Bond Elect (Dentsply), Optibond FL (Kerr), All-Bond 3 (Bisco), One Step Plus (Bisco), Scotchbond multi-purpose Adhesive (3M ESPE), Adper Easy One (3M ESPE), Gluma Comfort Bond (Heraeus), Gluma 2Bond (Heraeus) and Optibond S (Kerr); Clearfil SE Bond (Kuraray) and G-Aenial (GC Corp) were SE adhesives. Additionally, nine studies did not use any commercial adhesives, but used adhesives configured according to the experiment.

### Risk of bias

Based on the bias analysis parameters (Table 2), most of the studies had a low risk of bias in teeth free of caries/restoration (89.7%), and materials were used according to manufacturer’s instructions (89.7%). The majority of regarding teeth randomization (84.6) and sample size calculations were classified as having a medium risk of bias (84.6). Overall, the majority (88.5%) of them showed a moderate risk of bias, followed by low risk (7.7%) and high risk (3.8%). Furthermore, no studies reported blinding and only 11 (14.1%) reported single-operator adhesive procedures.

**TABLE 2:** Risk of bias of the studies considering aspects reported in the materials and methods section.

Study	Teeth randomization	Teeth free of caries/restoration	Materials used according to manufacturers’ instructions	Adhesive procedures performed by a single operator	Sample size calculation	Blind	Risk
Abd El-Aal et al. (2022)	YES	YES	YES	NO	YES	NO	Medium
Abdelshafi et al. (2021)	YES	YES	YES	YES	YES	NO	Low
Abunawareg et al. (2017)	YES	YES	YES	NO	YES	NO	Medium
Albuquerque et al. (2019)	YES	YES	YES	NO	YES	NO	Medium
Andre et al. (2015)	YES	YES	YES	NO	YES	NO	Medium
Bacelar-Sa et al. (2017)	NO	YES	YES	NO	YES	NO	Medium
Baena et al. (2020)	NO	YES	YES	NO	YES	NO	Medium
Baldion et al. (2021)	YES	YES	YES	NO	YES	NO	Medium
Beck and Ilie (2020)	YES	YES	YES	NO	YES	NO	Medium
Beck and Ilie (2022)	YES	YES	YES	NO	YES	NO	Medium
Carvalho et al. (2016)	YES	NO	YES	YES	YES	NO	Medium
Castellan et al. (2013)	NO	YES	YES	NO	YES	NO	Medium
Chen et al. (2021)	NO	YES	YES	NO	YES	NO	Medium
Chiang et al. (2013)	NO	YES	YES	NO	YES	NO	Medium
Comba et al. (2020)	YES	YES	YES	NO	YES	NO	Medium
Costa et al. (2019)	YES	YES	YES	NO	YES	NO	Medium
Cova et al. (2011)	YES	YES	YES	NO	YES	NO	Medium
Czech (2019)	YES	NO	YES	NO	YES	NO	Medium
Dacoreggio et al. (2021)	NO	YES	YES	NO	YES	NO	Medium
Daood et al. (2018)	NO	YES	YES	NO	YES	NO	Medium
Daood et al. (2018)	YES	YES	NO	NO	YES	NO	Medium
Daood (2020a)	YES	YES	YES	NO	NO	NO	Medium
Daood (2020b)	YES	YES	NO	NO	NO	NO	Low
de Macedo (2019)	YES	YES	NO	NO	YES	NO	Medium
de Paula (2022)	YES	YES	YES	NO	YES	NO	Medium
Dávila-Sánchez et al. (2020)	YES	NO	YES	NO	YES	NO	Medium
de Siqueira (2020)	YES	NO	YES	YES	YES	NO	Medium
De-Paula (2020)	NO	YES	YES	NO	YES	NO	Medium
Diolosa 2014	YES	YES	NO	NO	YES	NO	Medium
dos Santos (2018)	YES	YES	NO	NO	YES	NO	Medium
Du (2012)	YES	NO	YES	NO	YES	NO	Medium
Epasinghe (2015)	YES	YES	NO	NO	YES	NO	Medium
Fang et al. (2017)	NO	YES	YES	NO	YES	NO	Medium
Fawzy et al. (2012)	YES	YES	YES	YES	YES	NO	Low
Fawzy et al. (2013)	YES	NO	YES	NO	YES	NO	Medium
Fernandes (2021)	YES	YES	YES	NO	YES	NO	Medium
Fernandes (2022)	YES	YES	YES	NO	YES	NO	Medium
Fialho (2019)	YES	NO	YES	NO	YES	NO	Medium
Fonseca et al. (2019)	YES	YES	YES	NO	YES	NO	Medium
Fu et al. (2020)	YES	YES	NO	NO	YES	NO	Medium
Gerhardt (2016)	YES	YES	YES	NO	NO	NO	Medium
Gotti (2015)	YES	YES	YES	NO	YES	NO	Medium
Hass et al. (2016a)	YES	YES	YES	NO	NO	YES	Medium
Hass et al. (2016b)	YES	YES	YES	NO	YES	NO	Medium
Hechler (2012)	YES	YES	NO	NO	YES	NO	Medium
Jowkar (2020)	YES	NO	YES	NO	YES	NO	Medium
Lee (2017)	YES	YES	YES	YES	NO	NO	Medium
Li (2017)	YES	YES	YES	NO	YES	NO	Medium
Li (2018)	YES	YES	YES	NO	NO	NO	Medium
Li et al. (2021a)	YES	YES	YES	NO	YES	NO	Medium
Li et al. (2021b)	YES	YES	YES	NO	YES	NO	Medium
Li (2022)	YES	YES	YES	NO	YES	NO	Medium
Loguercio et al. (2017)	YES	YES	YES	YES	NO	NO	Medium
Magalhaes Rolim (2022)	YES	YES	YES	YES	YES	NO	High
Maravic et al. (2018)	YES	YES	YES	NO	YES	NO	Medium
Maravic et al. (2021)	YES	YES	YES	NO	YES	NO	Medium
Mazzoni (2013a)	YES	YES	YES	NO	YES	YES	Medium
Mazzoni et al. (2018)	YES	YES	YES	NO	YES	NO	Medium
Neri (2016)	YES	YES	YES	NO	YES	NO	Medium
Paik (2022)	YES	YES	YES	NO	YES	NO	Medium
Paludo (2019)	YES	YES	YES	NO	YES	NO	Medium
Paschoini (2021)	YES	YES	YES	NO	YES	NO	Medium
Paulose (2017)	YES	YES	YES	NO	YES	NO	Medium
Paulose (2018)	YES	YES	YES	NO	YES	NO	Medium
Porto (2018)	YES	YES	YES	NO	NO	NO	Medium
Santiago (2013)	YES	YES	YES	NO	YES	NO	Medium
Scheffel (2015)	YES	YES	YES	NO	YES	NO	Medium
Singh et al. (2015)	YES	YES	YES	NO	NO	NO	Medium
Sun (2018)	YES	YES	YES	NO	YES	NO	Medium
Venigalla (2016)	YES	YES	YES	NO	NO	NO	Medium
Yang (2016)	YES	YES	YES	YES	YES	NO	Low
Yang (2017)	NO	YES	YES	NO	YES	NO	Medium
Yu (2017)	YES	YES	YES	NO	YES	NO	Medium
Yu (2022)	YES	YES	YES	YES	YES	NO	Low
Zhang (2014)	NO	YES	YES	NO	NO	NO	High
Zhang (2016)	NO	YES	YES	NO	NO	NO	High
Zhao 2021	YES	YES	YES	YES	YES	NO	Low
Zheng and Chen, (2017)	YES	YES	YES	NO	YES	NO	Medium

### Meta-analyses

**FIGURE 3:**
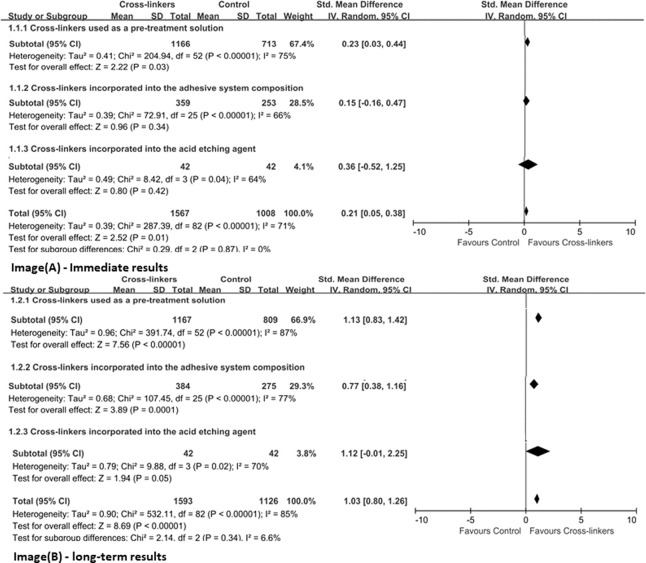
Effect of different application methods of cross-linkers.

Due to the heterogeneous distribution of adhesives used, a global meta-analysis of all 78 studies was not performed. Hence, cross-linkers were first assigned to the subgroups (immediate or long-term) based on their mode of application (used as a pre-treatment solution, incorporated in adhesive systems or in acid etching agent). Figure 3 represents the meta-analysis results on immediate and long-term bond strengths with different application methods of cross-linkers, respectively. Overall, there was a significant difference between the groups, showing evidence that the presence of cross-linkers produced superior resin–dentin bonds than the control group (*p* < 0.05). For immediate results, the mean differences between the experimental and the control groups were higher when used as a pre-treatment solution (*p* ≤ 0.03), but not used by incorporating it into adhesive systems and acid etching agent (*p >* 0.05). In terms of long-term bond strength, regardless of the application method, the groups presented better bond potentials (*p* ≤ 0.05), and the heterogeneity of the three analyzed sets was high (I^2^ ≥ 70%).

**FIGURE 4:**
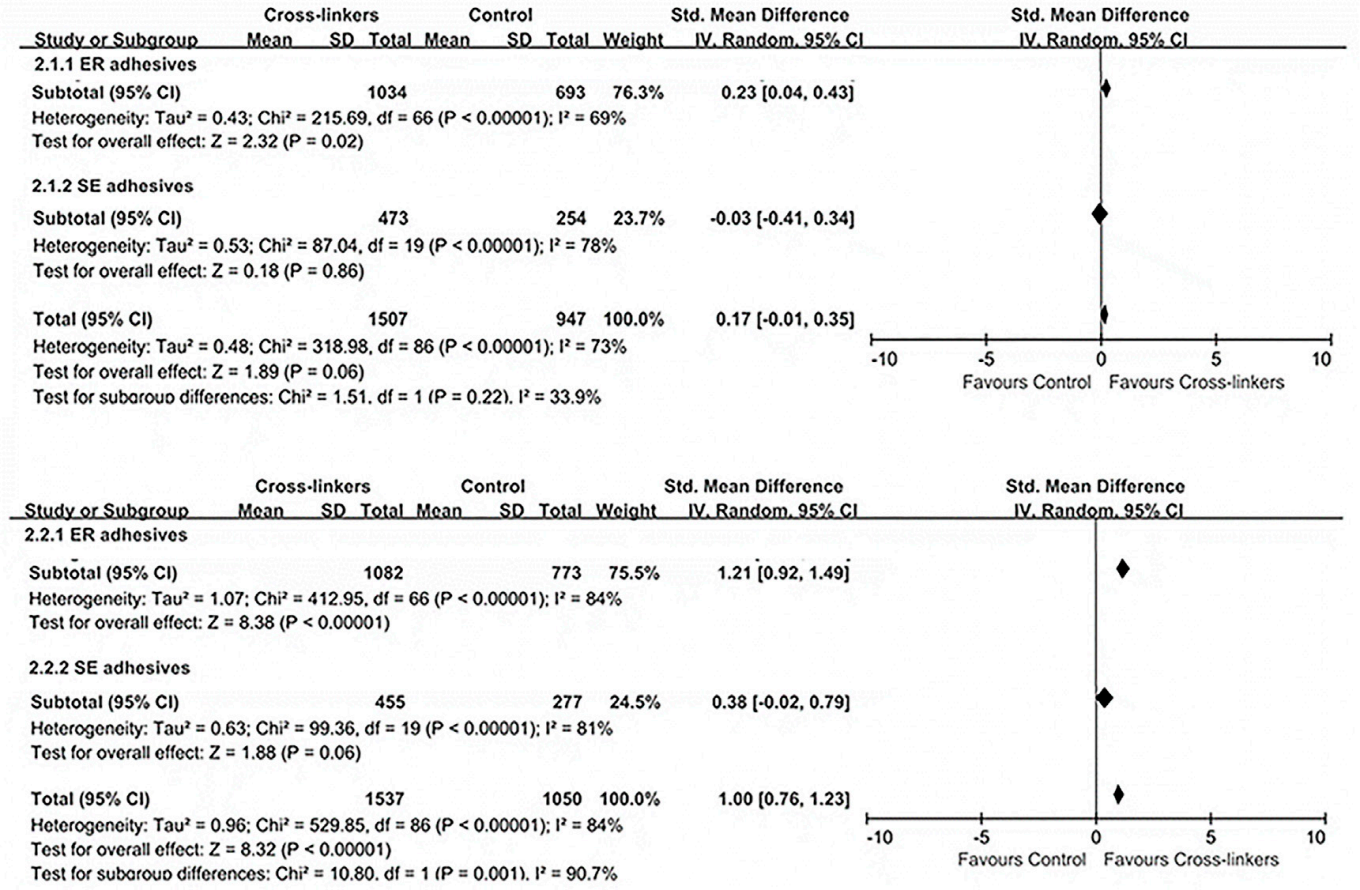
Effect of the application category of the adhesive system.

The meta-analysis on different adhesives used (Figure 4) showed significant differences between the groups using ER adhesives in favor of cross-linkers; both immediate (*p* ≤ 0.02) and long-term results (*p* < 0.00001) showed that the control group exhibited lower bonding potential than the experimental group. However, similar bonding effects were observed for SE adhesives between the cross-linkers and the control group (*p* > 0.05): immediate (effect size: 0.03, 95% CI: 0.41, 0.34; *p* = 0.86) and long-term results (effect size: 0.38, 95% CI: 0.02, 0.34; *p* = 0.79).

**FIGURE 5:**
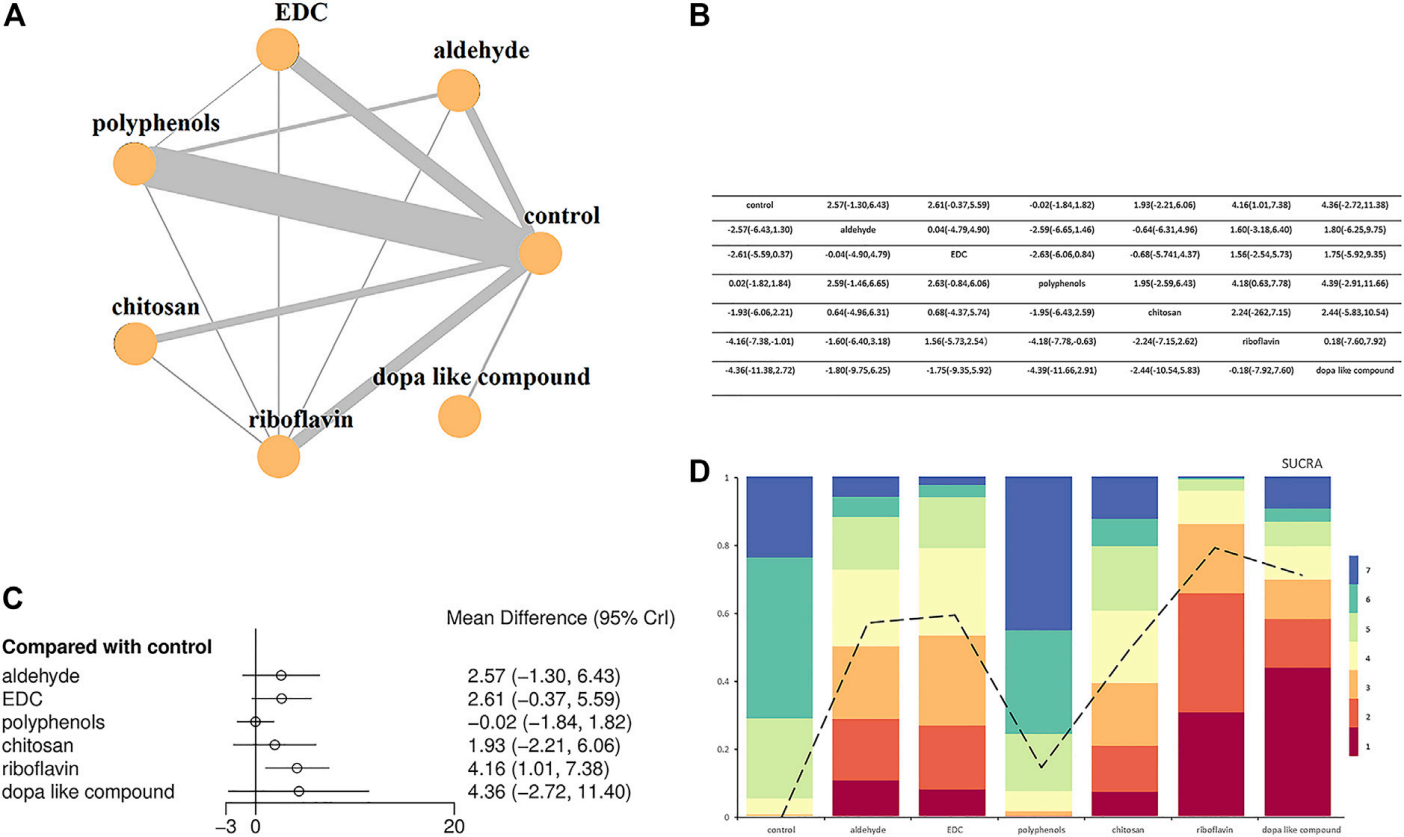
Network meta-analysis comparing bond strengths among seven group arms. **(A)** Network plot where each node indicates a direct comparison (control, aldehyde, EDC, polyphenols, chitosan, riboflavin and dopa like compound) with the thickness of connecting lines between nodes representing the number of studies compared. **(B)** League table showing Bayesian comparisons for all groups: this table shows the results for all group pairs in the upper (direct comparison) and lower (indirect comparison) triangles, but the comparisons have been switched; For the leading diagonal above and below, the result is the grouping at the top of the same column versus the grouping to the left of the same row. **(C)** Bayesian random effect consistency model forest plot of the pooled effects estimates of bond strengths expressed in mean difference (MD) and respective 95% credible intervals (95% CrI) for different adhesive groups compared with the control group. **(D)** Cumulative ranks and SUCRA-values.

**FIGURE 6:**
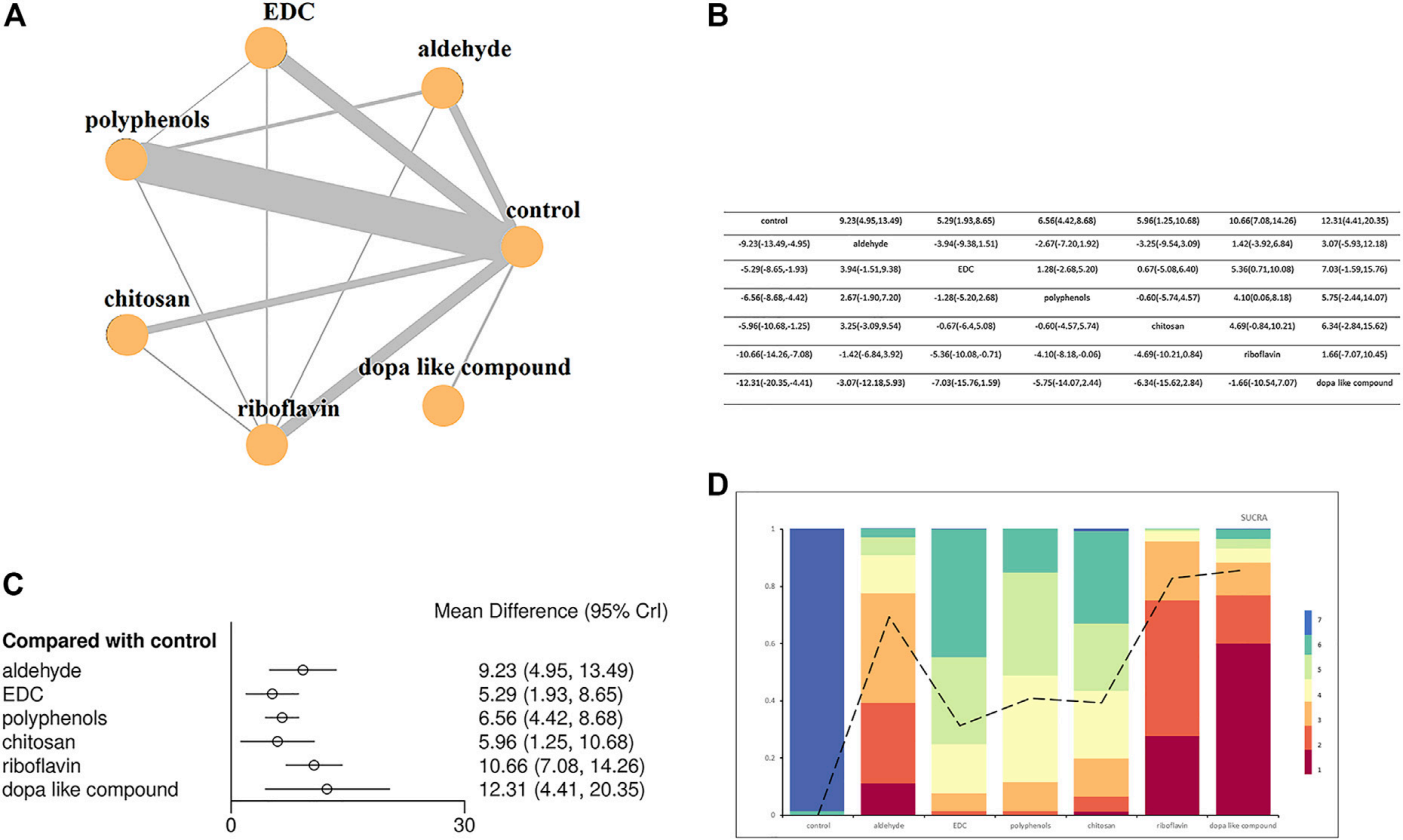
Network meta-analysis comparing the long-term bond strength between the control group and the six types cross-linkers. **(A)** Network plot where each node indicates a direct comparison (control, aldehyde, EDC, polyphenols, chitosan, riboflavin and dopa like compound) with the thickness of connecting lines between nodes representing the number of studies compared. **(B)** League table showing Bayesian comparisons for all groups: this table shows the results for all group pairs in the upper (direct comparison) and lower (indirect comparison) triangles, but the comparisons have been switched; For the leading diagonal above and below, the result is the grouping at the top of the same column versus the grouping to the left of the same row. **(C)** Bayesian random effect consistency model forest plot of the pooled effects estimates of bond strengths expressed in mean difference (MD) and respective 95% credible intervals (95% CrI) for different adhesive groups compared with the control group. **(D)** Cumulative ranks and SUCRA-values.

The NMA was conducted on studies grouped according to the presence or absence of as well as the type of cross-linkers (six categories), so a total of seven arms were compared with each other. Two sets of NMA were created, one for immediate (Figure 5) and the other for the long-term (Figure 6) data storage. Most pairwise comparisons were made between the “polyphenols” and the “control” groups (Figures 5A, 6A), while direct comparisons were made for all groups in the immediate and long-term subgroups, as shown in the league table (Figures 5B, 6B).

Forest plots comparing different types of crosslinkers with the “control” group demonstrated that the immediate bonding effect of crosslinkers and controls was similar, except for the “riboflavin” group (effect size: 4.16, 95% CrI: 1.01, 7.38) (Figure 5C), but long-term results showed that the former was associated with smaller resin-dentin bonding, with a reduction of 5.29–12.31 MPa (Figure 6C).

When evaluating the sorting probability and resulting SUCRA images (Figures 5D, 6D), riboflavin showed a higher immediate bond strength, while Dope like compound, EDC, aldehydes, chitosan, and polyphenols showed similar bonding performance as the control group (Figure 5B). In long-term outcomes, Dope like compound ranked highest, followed by riboflavin, aldehydes, polyphenols, chitosan, and EDC. The control group had the lowest ranking, while the possibility of smaller bonding properties was the highest, with limited differences (Figure 6D).

## Discussion

This is the first study to perform an NMA to compare the bonding performance of different adhesive systems to dentin, depending on the cross-linkers. The purpose of this study was to aee if the longevity lifespans of dentin bond strength could be effectively increased using a homogeneous systematic review. As much information was collected about the relevant studies to achieve reliability. In general, resin-dentin adhesion was favorable in the case of cross-linkers, depending on their types and application modes.

### Effect of different application methods of cross-linkers

In this meta-analysis, the majority of cross-linking agents were used as a pre-treatment solution (∼65.1%), followed by addition into the adhesive system (∼30.1%), and few were mixing in acid etching agents (4.8%) (Table 1). The analyses showed that the usage of cross-linkers as primers led to a significant improvement in the immediate and long-term bond strength values (*p* = 0.03 and *p* < 0.00001). The long-term bond strength was improved after adding cross-linkers to the adhesive system and acid etching agent (adhesive system, *p* = 0.0001; acid etching agent, *p* = 0.05).

It is already known that cross-linkers used alone as primers have gained popularity in the last two decades due to their superior crosslinking ability without compromising the degree of conversion of adhesives (Comba et al., 2020; Li et al., 2021a; Yu et al., 2022). Taking into consideration that cross-linkers were used only as a primer, they imparted greater bond strengths to dentin as compared to the control group (Figure 3). The disadvantage using cross-linkers as primers in clinical practice is that adds an extra step to the bonding protocol, making the process more difficult for the clinician (Liu et al., 2014). Incorporating cross-linking agents into the adhesive system or acid etching agent can significantly improve bonding durability and not impair the immediate result (Figure 3), which is due to the formation of a stable HL with dentin (45, 50–52). For example, the ability to react with the dentin matrix and the consequent impact on reducing proteolytic degradation, maintains the integrity of HL, thereby improving its longevity (53, 54). Notably, cross-linkers possess three desirable properties that may favor superior dentin bonds: 1) Maintain an elaborate collagen network and facilitate inter-diffusion of solvent and hydrophilic monomers; 2) Increase the hardness of demineralized dentin, thereby reducing the plasticizing effect of absorbed water; 3) Inhibit collagenase activity, resulting in a reduced in collagen biodegradation rate at the resin-dentin interface (Kishen et al., 2016).

### Effect of the application category of the adhesive system

Based on 78 studies included in this review, a total of 20 different types of cross-linkers were investigated (Figure 2), with Adper Single Bond 2 representing the most frequently reported adhesive material (∼35.9%). Most of the analyzed bond strength data were derived from ER adhesives (∼77%), followed by SE adhesives. In the case of ER adhesives, cross-linkers showed greater dentin bond strengths as compared to the control group (Figure 4), which may be due to some characteristics of ER adhesives that allow the formation of a strong HL with dentin (Pashley et al., 2011). However, the results of SE adhesives, the dentin bond strengths were similarly distributed between the experiment and the control groups (Figure 4). Another study has shown that cross-linkers are more effective in preservation and enzyme silencing when used with ER adhesives due to the process of dentin etching which allows the cross-linker to interact freely with collagen molecules, and substrate that has been degraded by matrix metalloproteinases (MMPs) (Mazzoni et al., 2006). Cross-linkers may still have an effect on the resin-sparse, water-rich collagen fibril layer at the bottom of HL during long-term storage (Maravic et al., 2021). Furthermore, in SE adhesives, the dentin tubules are blocked by the smear layer, and cross-linking agents significantly reduce the exposed MMPs activity and the cross-linking of exposed collagen fibrils; thus, presenting exposed dentinal tubules along with spare collagen fibrils. Therefore, they are not so effective in strengthening the collagen matrix (Singh et al., 2015; Maravic et al., 2021).

### Effect of the different types of cross-linkers

In the presented review, all bond strength data were divided into seven different groups aiming for an NMA. The groups varied in terms of the presence/absence of cross-linkers as well as their classification as control, aldehydes, EDC, polyphenols, chitosan, riboflavin and Dope like compound. Overall, cross-linkers favor longer lifespans of the resin-dentin bonds by stabilizing HL (Breschi et al., 2018; Cai et al., 2018). Additionally, Figures 5, 6 demonstrate the efficacy of Dope like compound and riboflavin when compared to the other classes, especially in long-term results. This is an important finding that highlights which cross-linkers should be used to prevent the degradation of HL, thus providing directions for future research.

According to the network analysis, Dope like compound achieved the highest ranking in terms of bond strength, both immediate and long-term. It was observed that cross-linking occurs between the catechol groups of MAP and amino groups of collagen fibrils through covalent bonds, which causes collagen to become stiff by preventing its triple-helix conformation from uncoiling (Sabatini and Pashley, 2014). Furthermore, MAP also enhances the resistance of the crosslinked collagen to enzymatic degradation, and can directly interfere with the active and the changed enzyme sites of the group (Fang et al., 2017). DMA consists of three different parts, namely carbon-carbon double bond, polyphenol structure, and connection group, made up of amide compound (Martinez Rodriguez et al., 2015). The carbon-carbon double bond can be combined with the grafting monomer. Whereas the two hydroxyl polyphenol groups are intersected with dentin collagen fibrils. Unlike the ester base, the amide base is more stable and provides durability in a moist environment (Rodrigues et al., 2015). As DMA can combine the adhesives and dentin as a whole unit, this unique feature allows it to enhance the strength of the collagen matrix stability and protect HL from hydrolysis (Li et al., 2020; Li et al., 2021a; Li et al., 2021b).

Riboflavin is a cross-linker producing free radicals *via* photooxidation (UVA), which improves the rigidity and mechanical stability of the collagen matrix, as well as the penetration capacity of the adhesive resin (Frassetto et al., 2016). Daood et al. proposed that the addition of riboflavin (0.1%) to the adhesive significantly increases the bond strength and maintains the resin-dentin bond’s durability without negatively affecting the degree of conversion (Fu et al., 2020). In the cross-linking mechanism of riboflavin, covalent bonds are formed within the collagen amino group (Zhang et al., 2011) and are cross-linked to proline and/or lysine in collagen *via* functional hydroxyl groups in riboflavin (Wollensak et al., 2007). It also inhibits MMPs activity, increases the stiffness of dentin collagen, and improves resin-dentin bonding (Cova et al., 2011; Chiang et al., 2013). Therefore, riboflavin is a relatively effective cross-linking agent, and this conclusion was consistent with the meta-analysis results.

It has been broadly accepted that GA performs better than other cross-linkers, since it increases type I collagen covalent bonds by cross-linking amino groups that bridge the lysine and hydroxylysine residues of different collagen polypeptide chains, as well as improves the mechanical properties of dentin, contributing to better dental bonds (54, 55). But its clinical application is limited due to its depolymerization effect and the high cytotoxicity of uncured molecules (47, 55). Recently, researchers have successively synthesized acrolein and FA, which, similarly to GA, bind to exposed collagen fibers, form stable covalent bonds, and produce intermolecular cross-links with adjacent collagen matrix (Maravic et al., 2018; Yu et al., 2022). Furthermore, they also inhibit the activity of collagenolytic enzymes in the deeper regions of HL, providing more efficacy than GA (Hass et al., 2016a). How to overcome GA’s cytotoxicity while ensuring an excellent cross-linking effect of the aldehyde group is a current research hotspot. Our results demonstrated that aldehydes could promote the bond strength of dentin.

The results suggest that polyphenols can effectively improve long-term bond strength without compromising immediate bond strength. Since the structure of natural polyphenols contains multiple phenolic hydroxyl groups, they enhance the structural stability of collagen molecules through hydrogen bonds. Several polyphenolic cross-linkers are now being used, among which PA and EGCG have the highest frequency. As a natural cross-linking agent, PA has antioxidant, antibacterial, and anti-inflammatory properties, and its low toxicity makes it a widely studied (Chen et al., 2022). A study has shown that using PA (6.5%) for dentin can promote long-term bond strength (Dávila-Sánchez et al., 2020). Moreover, PA provides a better collagen network and increased fiber volume, which enhance the penetration of adhesives and produce a higher-quality HL with greater bond strength (Zheng and Chen, 2017). Under clinically relevant circumstances, PA can effectively stabilize demineralized dentin collagen in anti-enzymatic activity, due to its non-covalent nature and covalent, electrostatic, and hydrophobic interactions with collagen molecules (Perdigão et al., 2013). Another study confirmed that strong bonds could be formed between the amide carbonyl group of collagen and the phenolic hydroxyl group of PA; resulting in the formation of proline-PA complexes (Parise Gré et al., 2018). Recent studies on the incorporation of PA into acid etching agents and experimental adhesives have demonstrated that they stabilize the dentin bonding interface without any adverse effects (Hass et al., 2016b; Loguercio et al., 2017).

It is well known that EGCG is a collagen cross-linker obtained from green tea with low toxicity and anti-inflammatory properties (Perdigão et al., 2013; Albuquerque et al., 2019), that stabilizes the collagen chain (Goo et al., 2003). It is worth mentioning that it reduces collagen biodegradation and increase the number of collagen crosslinks through hydrogen molecular interactions of acyl groups (Goo et al., 2003). This study outcomes are in accordance with previous studies that showed no adverse effects for the long-term bond efficacy to dentin, and displayed promoting effects without changing the degree of polymerization of experimental adhesives (Yu et al., 2017; Czech et al., 2019). Another cross-linker, chitosan, is a naturally hydrophilic polycationic biopolymer with inherent, adhesive potential and antibacterial properties, along with a wide range of dental applications (Shrestha and Kishen, 2012; Daood et al., 2013). Since chitosan displays properties of cross-linkage, a large number of free hydroxyl and amino groups form ionic complexes with collagen, which can produce microfiber arrangement in the collagen structure (Abd El-Hack et al., 2020). Notably, these cross-linked collagen matrices also possess antibacterial and anti-biofilm activities (Daood et al., 2018; Fonseca et al., 2019) The incorporation of chitosan into dentin adhesives increases long-term bond strength and creates an interface with antibacterial properties (Elsaka and Elnaghy, 2012; Diolosà et al., 2014) The usage of the chitosan-riboflavin combination enhanced the mechanical properties of dentin and synergistically reduced the degradation of the resin-dentin interface (Daood et al., 2018). The study results, in contrast to previous results (Hardan et al., 2022), refute the finding that chitosan is a weak crosslinker and does not have a significant effect when used alone.

According to thisanalysis, both the immediate and long-term bond strength levels improved after using EDC; it can be a good alternative to GA by forming amide bonds between the carboxyl and amino groups of collagen molecules. Moreover, it does not participate in the cross-linking process and shows greater biocompatibility due to urea derivatives (Nimni, 1988; Mazzoni et al., 2013a). In addition to the cross-linked collagen, EDC also interacts with the extracellular dentin matrix through MMPs inactivation by cross-linking the catalytic or non-catalytic parts of MMPS so that the substrate is unrecognized and gets cleaved, or the triple helix of the collagen molecule cannot be unwound (Mazzoni et al., 2017). EDC also cross-links proteins (collagen) by donating O-acyl urea groups, which activate the carboxylic acid groups of glutamate as well as aspartic acid peptide residues within 1-h treatment time and is clinically unacceptable (Cammarata et al., 2015). A recent *in vitro* study on EDC application on demineralized dentin for 60s suggested that EDC can persist in HL for 5 years, in term of bond strength, collagen structure preservation and dentinal enzyme silencing (Maravic et al., 2021). However, this might be relevant to the results of this study.

### Research prospects

The presented results showed that most of the studies focused on ER adhesives, while only a few the number of studies were on SE adhesives. Furthermore, the majority of the studies in this network analysis focused on control and polyphenols, followed by EDC and riboflavin. Due to its promising results, cross-linking can be considered a simple and clinically applicable method to improve bonding durability and reduce collagen degradation in HL. Currently, studies on various cross-linkers are still conducted in the laboratory, as it is difficult to simulate the challenges of the oral environment (pH, occlusal load and thermal stress, etc.). However, more clinical studies are needed to confirm the beneficial effects of these cross-linkers *in vivo*.

### Advantages and limitations of the study

This study has several advantages. It is the first study to investigate the effects of cross-linkers different factors on the bond strength of cross-linkers in dentin. The effects of different cross-linkers application methods and adhesive types on bond strength were analyzed by conventional meta-analysis, while the different types of cross-linkers were ranked by Bayesian analysis. However, this study also had some limitations. Since the heterogeneity was relatively large, it could have affected the accuracy.

## Conclusion

Due to a moderate heterogeneity in most studies based on this meta-analysis, an overall advantage of using cross-linkers for better dentine bond potential was observed. The results were dependent only on the application category of the adhesive system and were not affected by different application methods and the types of cross-linkers. The use of cross-linkers on acid-etched dentin increased the beneficial effects of cross-linkers and demineralized collagen and inhibited matrix metalloproteinases at the interface, which benefited dental bonding. Based on this meta-analysis, it is possible to conclude can be concluded that the application of different cross-linkers such as Dope like compound, riboflavin, GA, polyphenols, chitosan, and EDC improved the long-term bonding performance. It is worth noting that, of the cross-linkers examined in this review, Dope like compound have higher bonding potential to dentin than other classes of cross-linkers.

## Data Availability

The original contributions presented in the study are included in the article/Supplementary Material, further inquiries can be directed to the corresponding author.
