# Supplementary material for: Effect of collagen cross-linkers on dentin bond strength: A systematic review and network meta-analysis
Source: Front Bioeng Biotechnol. 2023 Jan 24;10:1100894. doi: 10.3389/fbioe.2022.1100894 (PMC9903368; doi:10.3389/fbioe.2022.1100894)
Supplement: Supplementary file 1 [file Table1.docx]

Supplementary Material 1-- Search strategy used in PubMed (MEDLINE).

| No.1 | (((((((((((((((((((((Cross-linker) OR (Cross-Linkers)) OR (Crosslinking)) OR (Crosslinking agents)) OR (Collagen cross-linkers)) OR (N,N’-dicyclohexylcarbodiimide)) OR (Carbodiimide)) OR (Ribose)) OR (Glutaraldehyde)) OR (Carbodiimide hydrochloride)) OR (Proanthocyanidins)) OR (Riboflavin)) OR (Chitosan)) OR (Polyphenols)) OR (Genipin)) OR (Flavonoids)) OR (4-formylphenyl acrylate)) OR (Epigallocatechin gallate)) OR (EGCG)) OR (condensed tannin)) OR (Hesperidin)) OR (Quercetin) |
| --- | --- |
| No.2 | (((((((((((Dental adhesive) OR (Dental adhesives)) OR (Dental bonding)) OR (Dentin bonding systems)) OR (Dentin bonding)) OR (dentine bonding)) OR (Universal adhesives)) OR (multi-purpose adhesives)) OR (multimode adhesives)) OR (universal bonding agent)) OR (Self-etch adhesives)) OR (Etch-and-rinse adhesives) |
| No.3 | ((((((((Bonding efficacy) OR (bond strength)) OR (Bonding performance)) OR (bonding effectiveness)) OR (Bond performance)) OR (adhesive properties)) OR (microtensile strength)) OR (Micro-tensile strength)) OR (bonding properties) |
| No.4 | No.1 AND No.2 AND No.3 |
